# Geopolymer/Zeolite composite materials with adsorptive and photocatalytic properties for dye removal

**DOI:** 10.1371/journal.pone.0241603

**Published:** 2020-10-30

**Authors:** Kedsarin Pimraksa, Naruemon Setthaya, Maneerat Thala, Prinya Chindaprasirt, Mitsuhiro Murayama

**Affiliations:** 1 Department of Industrial Chemistry, Faculty of Science, Chiang Mai University, Chiang Mai, Thailand; 2 Center of Excellence in Materials Science and Technology, Faculty of Science, Chiang Mai University, Chiang Mai, Thailand; 3 Division of Chemistry, School of Science, University of Phayao, Mae Ka, Phayao, Thailand; 4 Sustainable Infrastructure Research and Development Center, Faculty of Engineering, Khon Kaen University, Khon Kaen, Thailand; 5 Department of Materials Science and Engineering, Virginia Tech, Blacksburg, Virginia, United States of America; 6 The Virginia Tech National Center for Earth and Environmental Nanotechnology Infrastructure, Virginia Tech, Blacksburg, Virginia, United States of America; University of Southern Denmark, DENMARK

## Abstract

This study investigated the adsorption capacities and photocatalytic activities of geopolymer-zeolite composite materials by incorporating different amounts of zeolite and TiO_2_ in a geopolymer matrix for dye removal. Geopolymers with SiO_2_/Al_2_O_3_ molar ratio of 2.5 were synthesized from metakaolin. The geopolymers containing zeolite and TiO_2_-doped zeolite exhibited similar behavior in terms of mineral compositions, microstructures and chemical frameworks. The compressive strength of geopolymer-zeolite composite materials decreased with increasing amount of zeolite and TiO_2_-doped zeolite (0–40 wt%) because of the increase in the porosity of composite materials. The maximum methylene blue adsorption capacity and photocatalytic efficiency of the powdered geopolymer composites with 40 wt% TiO_2_-doped zeolite was 99.1% and was higher than that of the composites with 40 wt% zeolite without TiO_2_-doping (92.5%). In addition, the geopolymer composites with TiO_2_-doped zeolite exhibited excellent stability after repeated usage as photocatalysts. The adsorption capacity and photocatalytic activity of pelletized geopolymer composites decreased because of the reduction in their specific surface area.

## 1. Introduction

Dyes are globally used in many industries such as textile, leather, paper, automotive and printing. Therefore, colored sewage is generated in large quantities. Colored pollutants have severe hazardous effects on human health and environment [1–4]. The presence of dye compounds in aquatic environment can be extremely harmful to aquatic plants leading to large-scale destruction of ecosystem and life cycles [5]. Therefore, it is essential to remove dyes from wastewater prior to their discharge. Several techniques for the remediation of dye wastewater, including biological, physical and chemical processes such as membrane filtration, chemical flocculation-coagulation, and different types of oxidation and adsorption, have been developed for wastewater treatment [4, 6–8]. Among them, adsorption is an exceptionally efficient treatment for the removal of dyes as well as other organic and inorganic pollutants [7, 9]. In addition to activated carbon [10], zeolites have been widely used for adsorption to achieve the purification of dyes-contaminated wastewater because of their high pore volume, large exposed surface area to volume ratios and excellent adsorption capacity. Moreover, zeolites can be regenerated and reused several times [11, 12]. However, the application of zeolites limited because of the decrease in their adsorption capacity over long-term use, thereby hindering their utilization for dye adsorption. In addition, zeolite powder is not widely used because of the associated handling and disposal risks.

Recently, an ultraviolet (UV)-photooxidation process was demonstrated to have great potential to reduce organic pollution because it can completely degrade toxic substances [13, 14]. Titanium dioxide (TiO_2_) is considered as one of the most promising photocatalysts because of its excellent photocatalytic activity, chemical and biological inertness, nonphotocorrosive nature, thermal stability, low cost and nontoxicity properties. Therefore, TiO_2_ has been commonly used to eliminate pollutants from wastes photochemical solutions [15–18]. Because the concentration of pollutants in water and air is low and the adsorption ability of TiO_2_ is poor, its performance is limited [15]. One feasible ways to overcome this issue is to hybridize zeolitic materials with TiO_2_ photocatalyst in order to improve adsorption ability of TiO_2_. Therefore, this study focuses on the synthesis of geopolymer-based composite materials for dye adsorption. We also propose an alternative way to incorporate TiO_2_ containing zeolites in geopolymers to produce solidified materials that can be conveniently handled before and after use.

Synthetic zeolites are commonly produced by mixing solutions of silicates and aluminates, in order to form an aluminosilicate gel and finally crystallized zeolite. To precipitate the zeolite, a temperature of 100°C or more must be maintained in the aluminosilicate mixture for a certain period [19]. Zeolites are also found in geopolymers, particularly at the curing temperature of > 85°C; the zeolite amount increased with curing time [20, 21]. The chemical compositions of geopolymers are relatively similar to zeolites; however, a geopolymer possesses an amorphous microstructure that leads to several different properties. Geopolymers are formed by the copolymerization of aluminate and silicate species, generated from the dissolution of silicon- and aluminum-containing starting materials using a high pH condition and in the presence of an alkali silicate solution [22–24]. Geopolymerization includes a rapid chemical reaction involving aluminosilicate minerals, under alkaline condition, that results in framework structures, polymer chains and ring structures depending on the Si/Al ratio [25–27]. With alkaline activation, solidification can be obtained at low temperature (< 100°C), resulting in a material with binding properties. Geopolymer production is considerably more advantages in terms of energy consumption and production of greenhouse gas emission compared to the production of ordinary Portland cement. Various geopolymers or geopolymer-zeolite composite materials with unique characteristics have been widely fabricated for dye removal [28–34]. Zeolites exhibit high adsorption capacity with a unique microporous structure while geopolymer has mesoporosity [20, 35]. Zeolite-geopolymer composite materials, therefore, exhibit large pore size distribution. Each zeolite type displays unique pore characteristic that can be used for the encapsulation of organic dye molecules. For example, faujasite zeolite, with an average pore size of 1.36 nm, is appropriate for the physical adsorption of methylene blue (MB), a dye with an effective molecular size of ~0.77 nm [36, 37]. The mechanism of adsorption proceeds by cation exchange between cations of the zeolite and cationic MB molecules. In addition to cation exchange, MB molecules are adsorbed onto the surfaces of the zeolite. Moreover, geopolymer plays a crucial role in the solidification of zeolite powders, thus, geopolymer-zeolite composites afford trouble-free handling compared to the powder form of only zeolite. In addition, the collection after service life can be performed with minimal effort. Accordingly, geopolymer-zeolite composites with beneficial properties can be used as building materials with exceptional humidity control. This property is remarkable and worthy of in-depth investigation in the field of dye adsorption. Furthermore, geopolymer-zeolite composites doped with TiO_2_ have not been extensively studied.

The main objectives of this work were to fabricate TiO_2_-containing geopolymer-zeolite composite materials. TiO_2_-doped zeolites synthesized in a previous study [36] were used as a composite and metakaolin (MK) was employed as a starting material for geopolymer synthesis. The phase development, microstructures, and mechanical properties of the composite materials were investigated. The dye removal efficiency was evaluated by determining the adsorption and photocatalytic activities of the composites using MB (C_16_H_18_N_3_SCl) in water as a representative dye. The investigated geopolymer-zeolite composite materials were prepared in 2 forms for comparison purposes: powder and pellet. The pelletized composite materials were fabricated to allow alacritous collection after use. Moreover, the regeneration of the spent composites was examined to ascertain their reusability. This study lay a foundation for the sustainable control of contaminated systems.

## 2. Materials and methods

### 2.1 Materials

**Fig 1:**
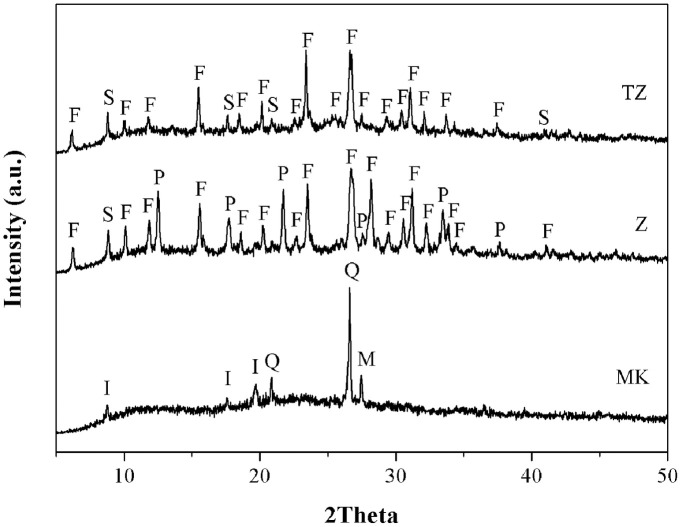
XRD patterns of starting materials. I; Illite, Q; Quartz, M; Microcline, F: Faujasite, P: Zeolite P1, S: Zeolite SSZ16.

**Fig 2:**
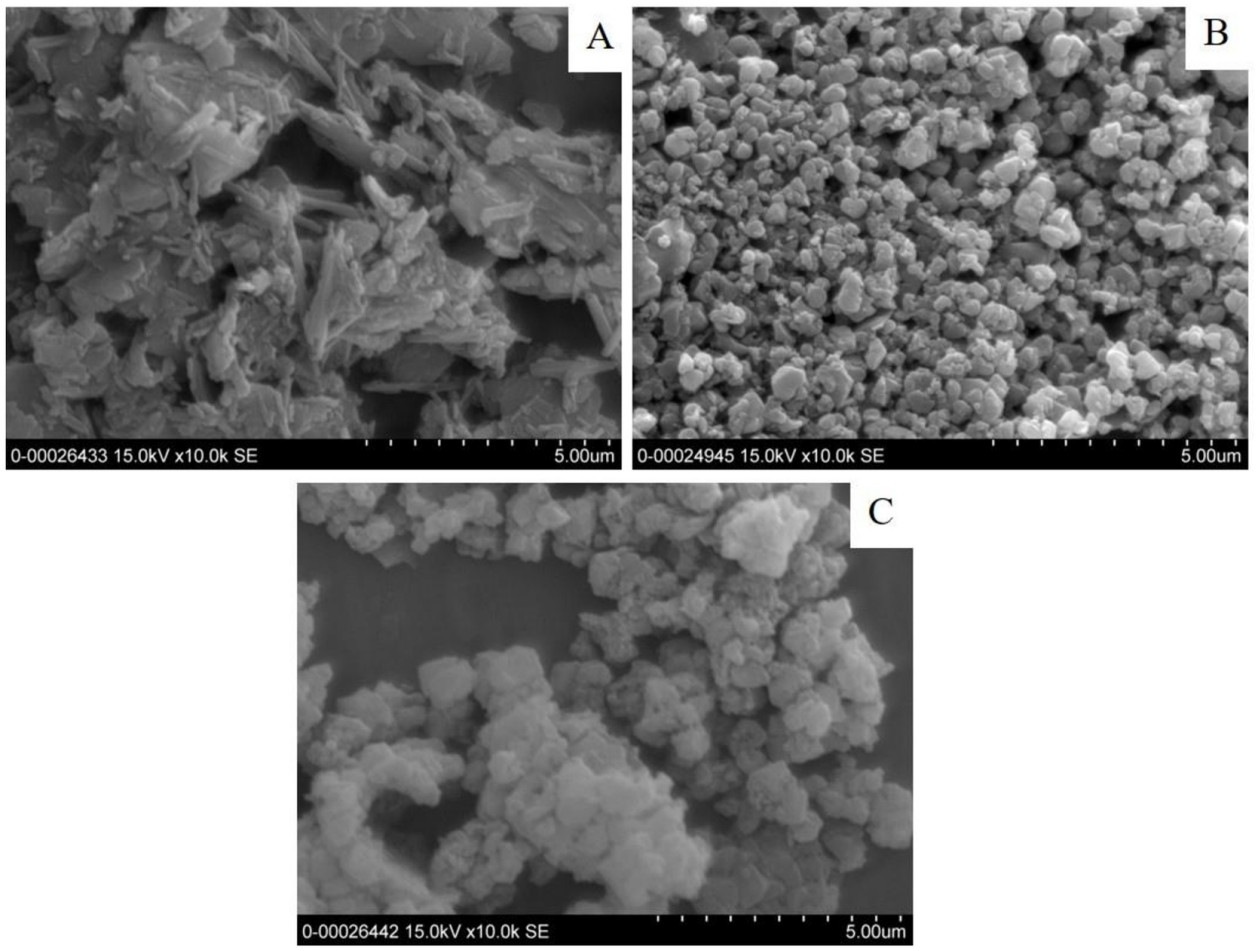
FE-SEM photomicrographs of materials (A) MK, (B) zeolite (Z) and (C) TiO_2_/zeolite (TZ).

Kaolin clay was purchased from Sibelco Mineral (Thailand) Co., Ltd. The calcination of kaolinitic clay was carried out at 750°C for 3h in an oxidizing atmosphere with a heating rate of 5°C/min [37]. The product was then ground and passed through a sieve (No.230; pore sizes < 63 μm). Zeolite (Z) was solvothermally synthesized using rice husk ash (RHA) and MK as the starting materials. The TiO_2_-doped zeolite composite (TZ) was obtained by the impregnation method using the synthesized zeolite and titanium (IV) butoxide (Ti(OC_4_H_9_)_4_). The zeolite and titanium source are similar to those utilized in a previous study [36]. The raw mix was prepared by mixing RHA and MK in a NaOH solution containing 10% ethanol with a SiO_2_/Al_2_O_3_ molar ratio of 4.0. The chemical compositions, mean particle size, and specific surface area of MK, Z, and TZ were characterized through X-ray fluorescence (Philips Magix Pro), Zetasizer–based nanoparticle analysis (Malvern Zetasizer Nano ZS), and Brunauer–Emmett–Teller (BET) analysis (Quantachrome Autosorp-1), respectively. The results of these analyses are shown in Table 1. The mineralogical compositions and morphology were observed through X-ray diffraction analysis (XRD, Bruker AXS D2 Phaser) and field-emission scanning electron microscope (FE-SEM, SU-6600, Hitachi) and are shown in Figs 1 and 2, respectively. Sodium hydroxide (97% NaOH, Wako Pure Chemical Industries, Ltd.) and sodium silicate solution (36.50% SiO_2_, 18.00% Na_2_O, Kanto Chemical Co., Inc.) were used as alkaline activator for geopolymer synthesis.

**Table 1:** Characteristics of the materials.

Characteristics	Metakaolin (MK)	Zeolite (Z)	TiO_2_-Zeolite (TZ)
1. Chemical compositions			
SiO_2_	53.60	46.23	45.67
Al_2_O_3_	40.60	24.60	23.19
CaO	0.70	0.80	0.87
K_2_O	1.92	1.24	1.28
Na_2_O	0.08	11.21	11.30
Fe_2_O_3_	1.64	1.14	1.05
P_2_O_5_	0.03	0.25	0.09
TiO_2_	0.08	0.05	6.53
MgO	0.56	0.51	0.59
LOI	0.75	13.76	9.85
2. Mean particle size (μm)	2.75	0.62	0.71
3. Specific surface area by BET (m^2^ g^-1^)	21.16	487.10	437.60

### 2.2 Synthesis and characterization

The geopolymers were synthesized using sodium silicate and 10 M sodium hydroxide solutions, with SiO_2_/Al_2_O_3_ molar ratio of 2.5 and Na_2_O/Al_2_O_3_ molar ratio of 1.1 [21, 38]. In addition to MK, 10%, 20%, 30%, and 40 wt% of Z and TZ powders were used. The mixtures were dryly mixed in ball mill for 30 min and then in the alkaline solution for 5 min to obtain a uniform mixture. Subsequently, the pastes were cast into a 25 mm^3^ acrylic cube mold. The mold was then wrapped with a plastic film to prevent loss of moisture. After a delay time of 1 h in a temperature-controlled room (25°C), the samples were cured at 60°C in an electric oven for 48 h and allowed to cool to room temperature before demolding. The samples were stored at 25°C and 50% relative humidity. The specimens were tested for compressive strengths at the age of 7 days in accordance with ASTM C109 standard. Samples containing 40 wt% Z and TZ were also tested for water absorption after 24 h immersion in water. The percentage of water absorption is defined as the difference between the weight of the sample submerged in water 24 h and dry weight of the sample.

The observation of the morphologies and elemental analyses were conducted on the surfaces of the samples that were fractured after the compressive strength test through FE-SEM (SU-6600, Hitachi) and energy dispersive X-ray spectrometer (EDS, Inca x-act, Oxford Instruments), respectively. The samples were ground to obtain a mean particle diameter of 45.25 μm for XRD analysis using a Bruker AXS D2 Phaser with graphite-monochromized Cu Kα radiation. Fourier transform infrared (FTIR) spectroscopy was performed using a PerkinElmer Spotlight 400 imaging system. The area under the peak was determined using the Origin data analysis program.

### 2.3 Testing for methylene blue removal efficiency and photocatalytic performance

The removal efficiency and photocatalytic degradation of MB by the synthesized geopolymer composites were determined through 2 experiments. For the first experiment, geopolymer composites in which 0–40 wt% TZ was added were ground to a powder with a mean particle diameter of 235.45 μm. MB removal and photodegradation were carried out in a 250 mL reactor. The second experiment was carried out using geopolymer composite pellet (15 x 15 x 21 mm^3^) with 30 wt% TZ to determine the recovery after use. With the addition of 40 wt% TZ, the homogeneity of the mixture decreased and the material was difficult to shape. A UV high-pressure lamps with E27 base (Osram Ultra Vitalux 300 W) operating over a wavelength range of 300–365 nm was used as a light source. The initial concentration of MB was determined by measuring its maximum absorbance (λmax = 664 nm) using a Hewlett-Packard 8453 UV-Visible spectrophotometer. Prior to illumination, a suspension containing 0.2 g ground geopolymer composite and 100 mL MB solution (40 mg/L) was continuously stirred at 500 rpm in a dark chamber for 30 min to attain adsorption equilibrium. The illuminated suspension was then collected at regular time intervals and centrifuged at 3000 rpm to obtain a clear solution. The geopolymer composite pellet was tested in a similar way. Geopolymer composite pellet (10 g) were soaked in 100 mL of MB solutions (30 and 40 mg/L) in a 250 ml reactor. This experiment was performed without stirring. The MB removal efficiency was also monitored at a wavelength of 664 nm using UV-Visible spectrophotometer. The MB removal efficiency *(X)* was calculated using the following equation:

$$X=[({\text{C}}_{0}–{\text{C}}_{\text{t}})/{\text{C}}_{0}]\;\text{x}\;100$$

where C_0_ is the initial concentration of the MB solution and C_t_ is the concentration of MB after adsorption under dark condition and photodegradation upon irradiation analyzed at time *t*.

## 3. Results and discussions

### 3.1 Characterization of starting materials

The chemical compositions and physical properties of the MK, Z and TZ are shown in Table 1. The silica and alumina contents of MK were 53.60 and 40.61wt%, respectively corresponding to a SiO_2_/Al_2_O_3_ molar ratio of 2.24. The SiO_2_/Al_2_O_3_ molar ratio obtained for Z was close to that for TZ (3.2–3.3). For TZ, the TiO_2_ content was 6.53 wt%. The mineralogical compositions of the starting materials are shown in Fig 1.

MK consisted of quartz (SiO_2_), illite (KAl_3_Si_3_H_2_O_12_), microcline (KAlSi_3_O_8_) and amorphous aluminosilicate from the dehydroxylation of kaolinite mineral. Faujasite (Na_2_Al_2_Si_3.3_O_10.6_(H_2_O)_7_) and zeolite P1 (Na_6_Al_6_Si_10_O_32_(H_2_O)_12_) are the major and minor crystalline phases, respectively in the synthesized zeolites (Z). For TZ, similar to Z, faujasite was observed; however, TZ contained different minor phases of zeolite SSZ16. The diffraction peaks of TiO_2_ phase were not detected for TZ. The mean particle size of MK, Z and TZ were 2.75, 0.62 and 0.71 μm, respectively. The specific surface area of MK, Z and TZ were 21.16, 487.10 and 437.60 m^2^/g. Z and TZ possessed relatively high specific surface area because of their porous nature. The morphologies of MK, Z and TZ are shown in Fig 2, indicating that MK particles contained stacked plate-like particles. The particle shapes of Z were similar to those of TZ because of their identical major phase that comprised various sizes of agglomerated octahedral particles.

The zeolites (either with or without doped TiO_2_) synthesized with MK and RHA had SiO_2_/Al_2_O_3_ molar ratio of 3.2–3.3. The addition of RHA and MK increased the SiO_2_/Al_2_O_3_ ratio of the starting materials to 4.0 because of the highly siliceous nature of RHA. This indicates that not all SiO_2_ present in RHA or MK played a role in the precipitation of zeolites. Only active silica formed aluminosilicate structures such as faujasite and zeolite P1. It was confirmed that the employed zeolites were the same type either with (TZ) or without the addition of TiO_2_ (Z) as demonstrated by the XRD patterns in Fig 1. The TiO_2_ clusters formed after the heat treatment step were too small to exhibit clear diffraction patterns [36, 39]. The dissolution-precipitation of zeolites facilitated the formation of smaller particles compared to the particle size of the starting materials. Their specific surface areas were also significantly larger because of the highly porous nature of zeolitic materials.

### 3.2 Characterization and testing of geopolymer composite materials

#### 3.2.1 Mineralogy

**Fig 3:**
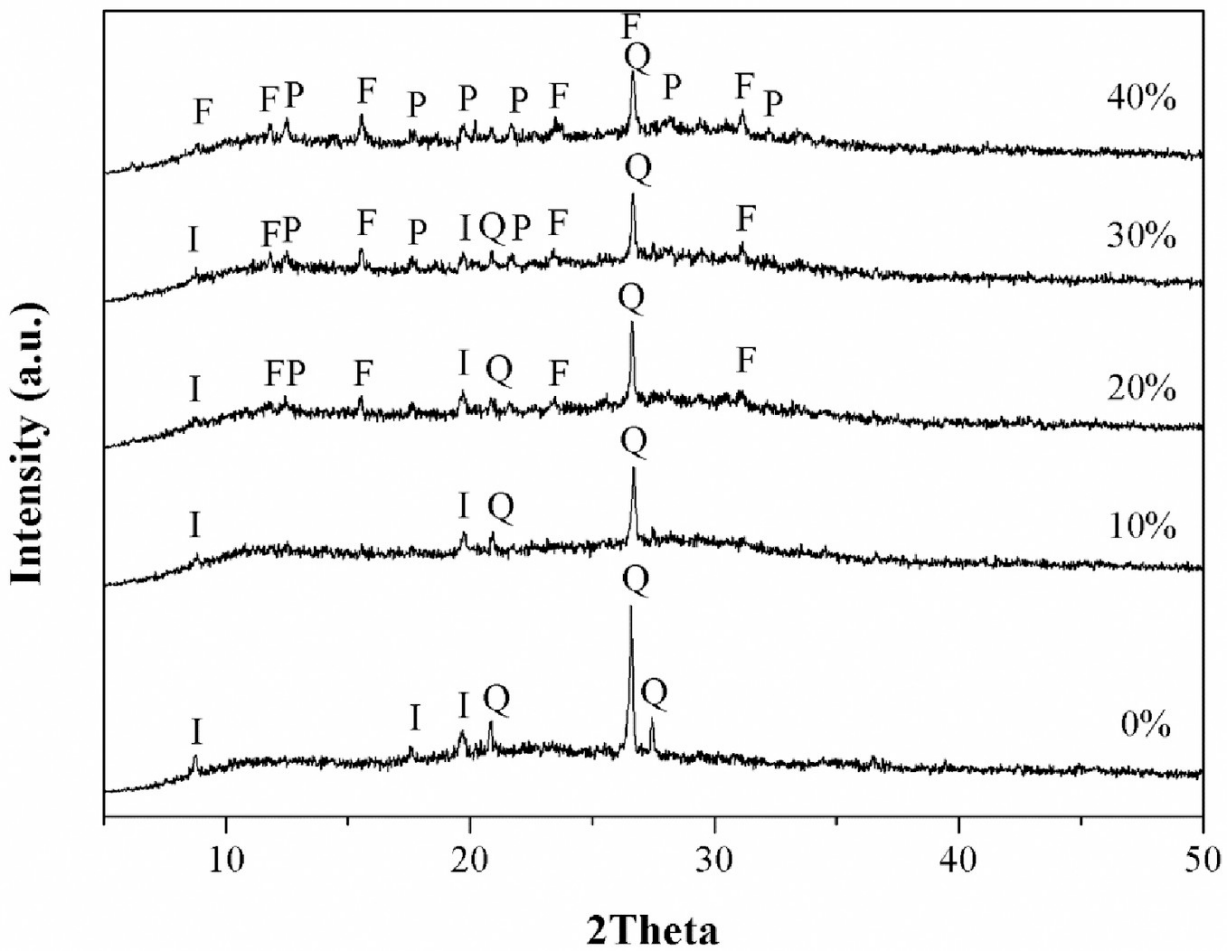
XRD patterns of MK-based geopolymer containing different amounts of zeolite. I; Illite, Q; Quartz, F: Faujasite, P: Zeolite P1.

XRD patterns of MK based geopolymers containing different amounts of Z (0 to 40 wt%) are shown in Fig 3. The XRD patterns showed that the MK-based geopolymer with 0 and 10 wt% Z contained unreacted minerals found in MK (quartz and illite) and amorphous geopolymeric material.

**Fig 4:**
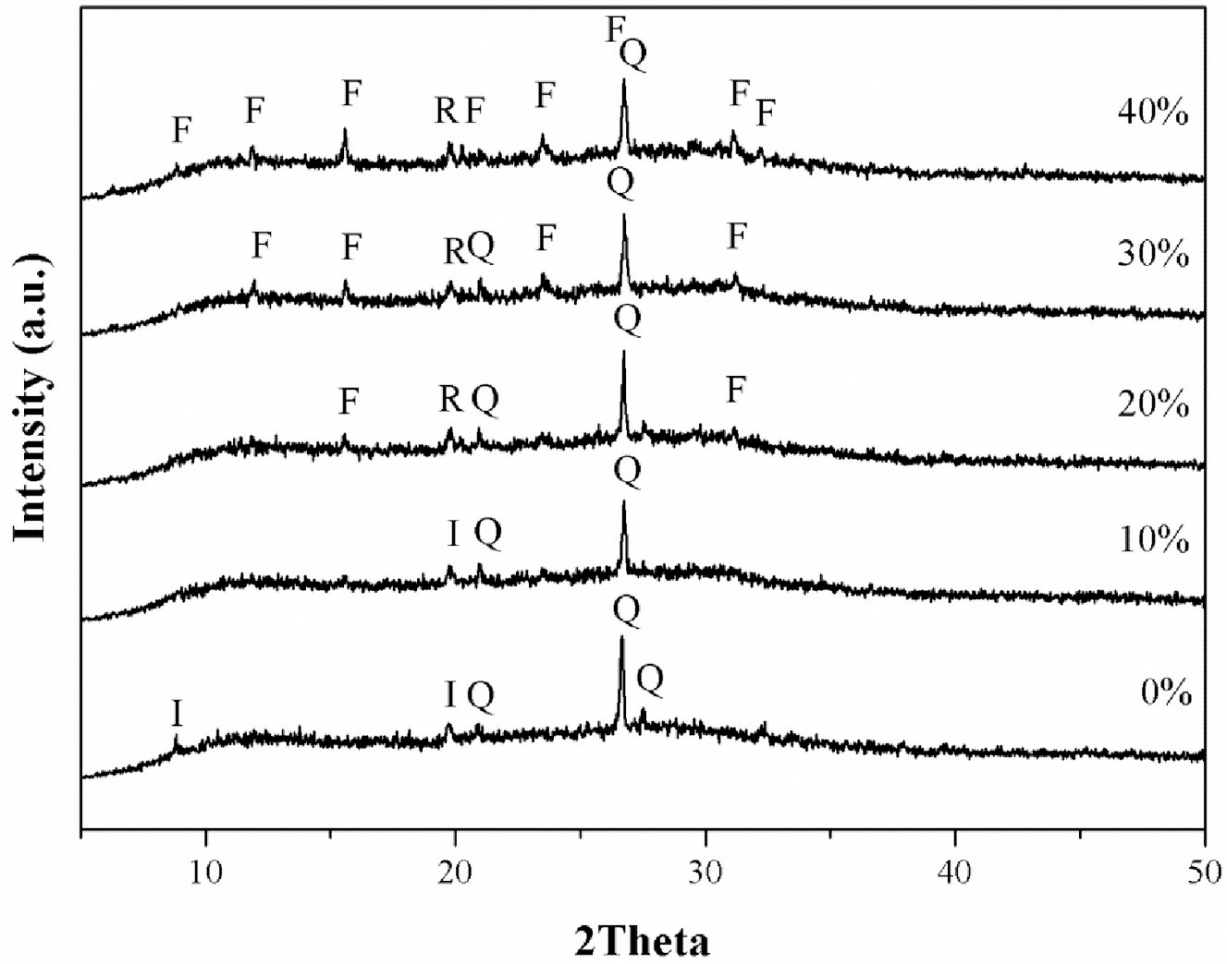
XRD patterns of MK-based geopolymer containing different amounts of TiO_2_/zeolite. I; Illite, Q; Quartz, F: Faujasite, R: Zeolite Rho.

No zeolite was found for MK-based geopolymers with low amount of Z. The additions of 20 and 30 wt% Z resulted in a reduction of the quartz content because of the dilution effect and the presences of faujasite and zeolite P1 phases. For the MK-based geopolymers with 40 wt% Z, illite and small amount of quartz disappeared with the emergence of the faujasite and zeolite P1. The peak intensity of faujasite and zeolite P1 increased compared to that of geopolymers with 20 and 30 wt% Z. Fig 4 shows the XRD patterns of MK based geopolymers with different amounts of TZ.

For geopolymers with0 and 10 wt% TZ, results similar to those with 0 and 10 wt% Z were observed. It was worth noting here that a new phase of zeolite Rho (H_12_[Al_12_Si_36_ O_96_]) was present in the geopolymers with 20 to 40 wt% of TZ, while zeolite SSZ16 disappeared. Moreover, for the geopolymers with 40 wt% TZ, quartz was transformed to faujasite zeolite; however, a small amount of quartz may remain partially intact. Thus, the appearance of faujasite zeolite in the geopolymer obtained with Z and TZ and the geopolymerization process in MK based geopolymer with 20–40% Z or TZ did not affect the zeolite transformation. The disappearance of Z upon 10 wt% Z addition, as shown in the XRD-patterns (Fig 3) was attributed to the dissolubility of Z in high concentration alkaline solution [40, 41]. Therefore, the dissolved SiO_2_ and Al_2_O_3_ played a role in the condensation of the geopolymer. The additions of 20 and 30 wt% Z resulted in a reduction of the quartz content that was also attributed to the dilution effect and the presences of faujasite and zeolite P1 phases. This indicated that the dissolution of zeolites in an alkaline solution was limited. With 10 wt% addition, TZ dissolved in the alkaline solution, similar to the case of Z addition because of their common zeolitic phases, as shown in Fig 4. The new Rho phase was found in the geopolymer composites with 20–40 wt% TZ. It was worth noting here that zeolite SSZ16 was unstable in alkaline solutions and transformed to zeolite Rho. In addition, the SiO_2_/Al_2_O_3_ molar ratios chosen for geopolymer synthesis in this study favored the formation of a zeolitic phase [42]. However, with 20–40 wt% addition of either Z or TZ, geopolymer composites contained a faujasite phase, which is a porous material suitable for dye adsorption.

#### 3.2.2 Microstructures

**Fig 5:**
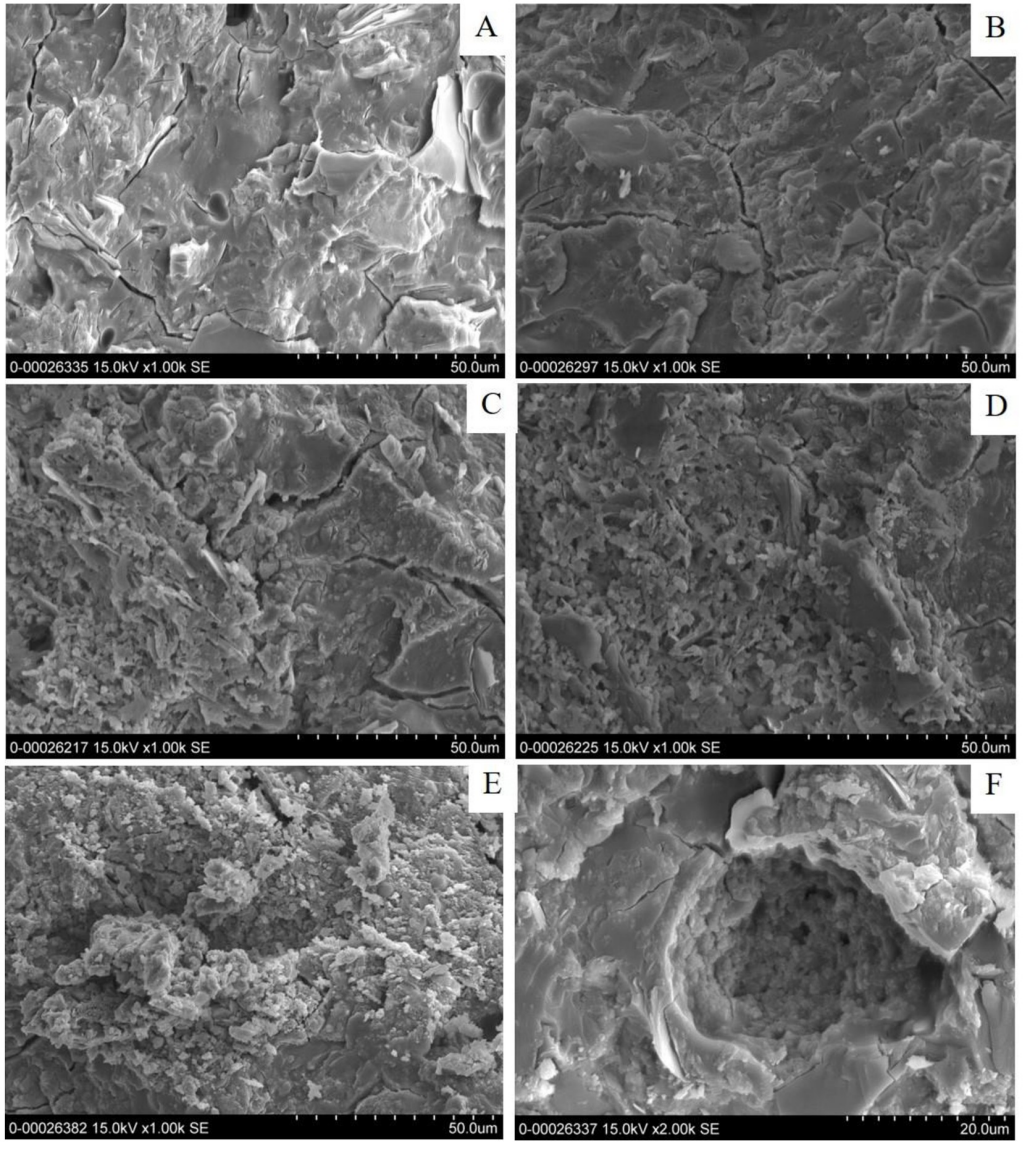
FE-SEM photomicrographs of MK-based geopolymer containing different amounts of zeolite (A) 0%, (B) 10%, (C) 20%, (D) 30%, (E) and (F) 40 wt%.

The FE-SEM photomicrographs of the synthesized MK-based geopolymer with Z addition are shown in Fig 5. These demonstrated that the polymerization of geopolymeric gel produced well-connected, glassy phase structures with no grain boundaries. Without or with a low Z content of 10 wt%, the matrices of MK-based geopolymer appeared as dense microstructures, as shown in Fig 5A and 5B.

**Fig 6:**
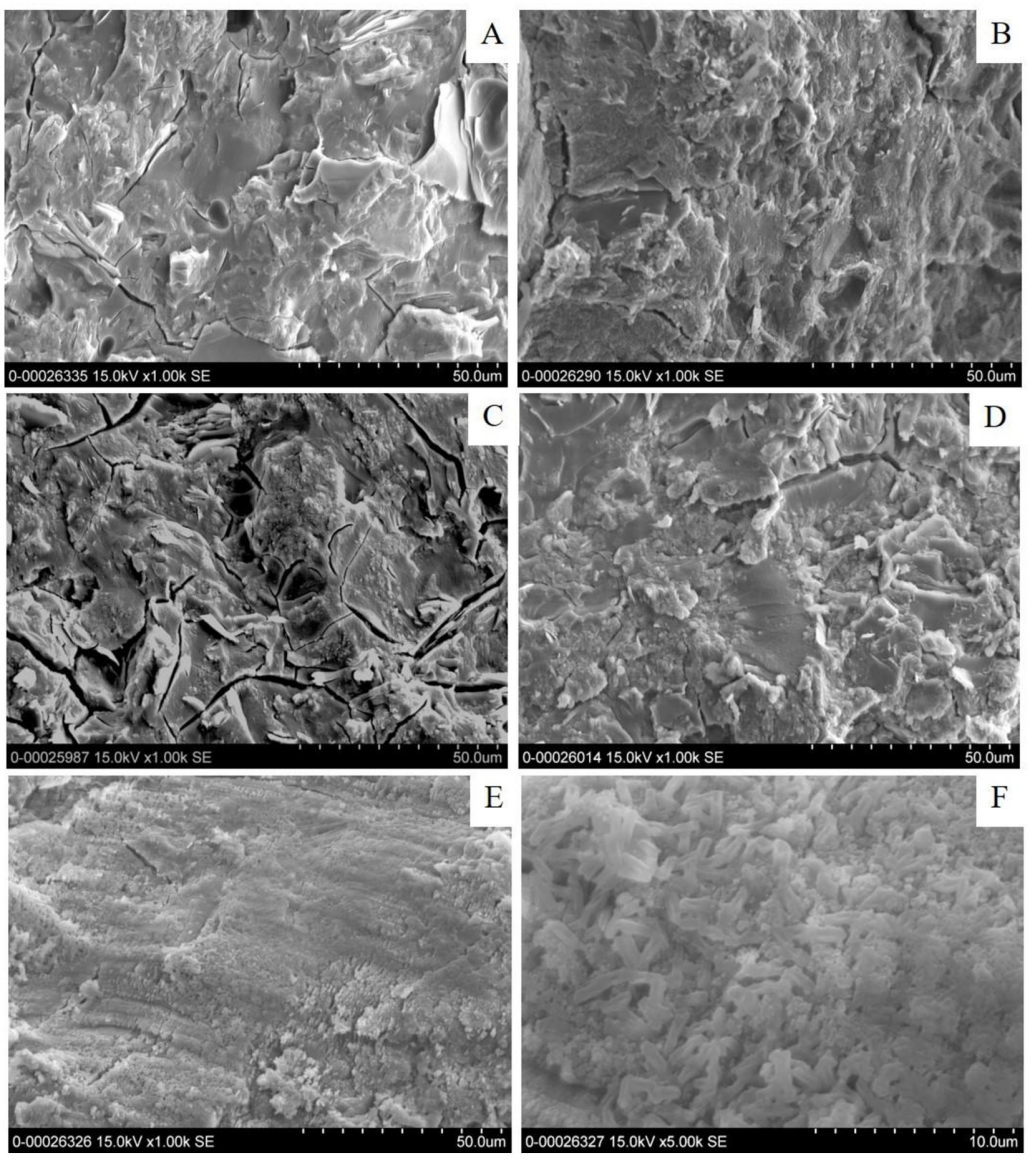
FE-SEM photomicrographs of MK-based geopolymer containing different amounts of TiO_2_/zeolite (A) 0%, (B) 10%, (C) 20%, (D) 30%, (E) and (F) 40 wt%.

As mentioned previously, the dissolved zeolite species can be present in the geopolymeric structure, introducing excellent connectivity among the geopolymer phases. Therefore, no composite material containing 10 wt% Z was prepared. For the geopolymers with 20, 30 and 40 wt% Z, the matrices were less dense as shown in Fig 5C–5F, because of the presence of faujasite zeolite as a composite material. It can be clearly seen in Fig 5F that the internal matrices of MK-based geopolymer with 40 wt% Z contained large zeolite particles belonging to the faujasite type (starting materials). This result was in a good agreement with the XRD patterns in which the zeolite starting materials remained intact. In a similar way, the geopolymers with 0 and 10 wt% TZ appeared as extremely dense matrices of the MK-based geopolymer and sparse matrices for the higher amounts of TZ (20–40 wt%), as shown in Fig 6A–6F. For the higher amount of TZ (20–40 wt%), shown in Fig 6C–6F, the density and the homogeneity of the matrices were reduced. Fig 6F clearly demonstrated that the crystalline zeolitic particles were present in the matrices. The limit for zeolite dissolution upon Z and TZ incorporation in these alkaline environments was, therefore, 10 wt%.

**Fig 7:**
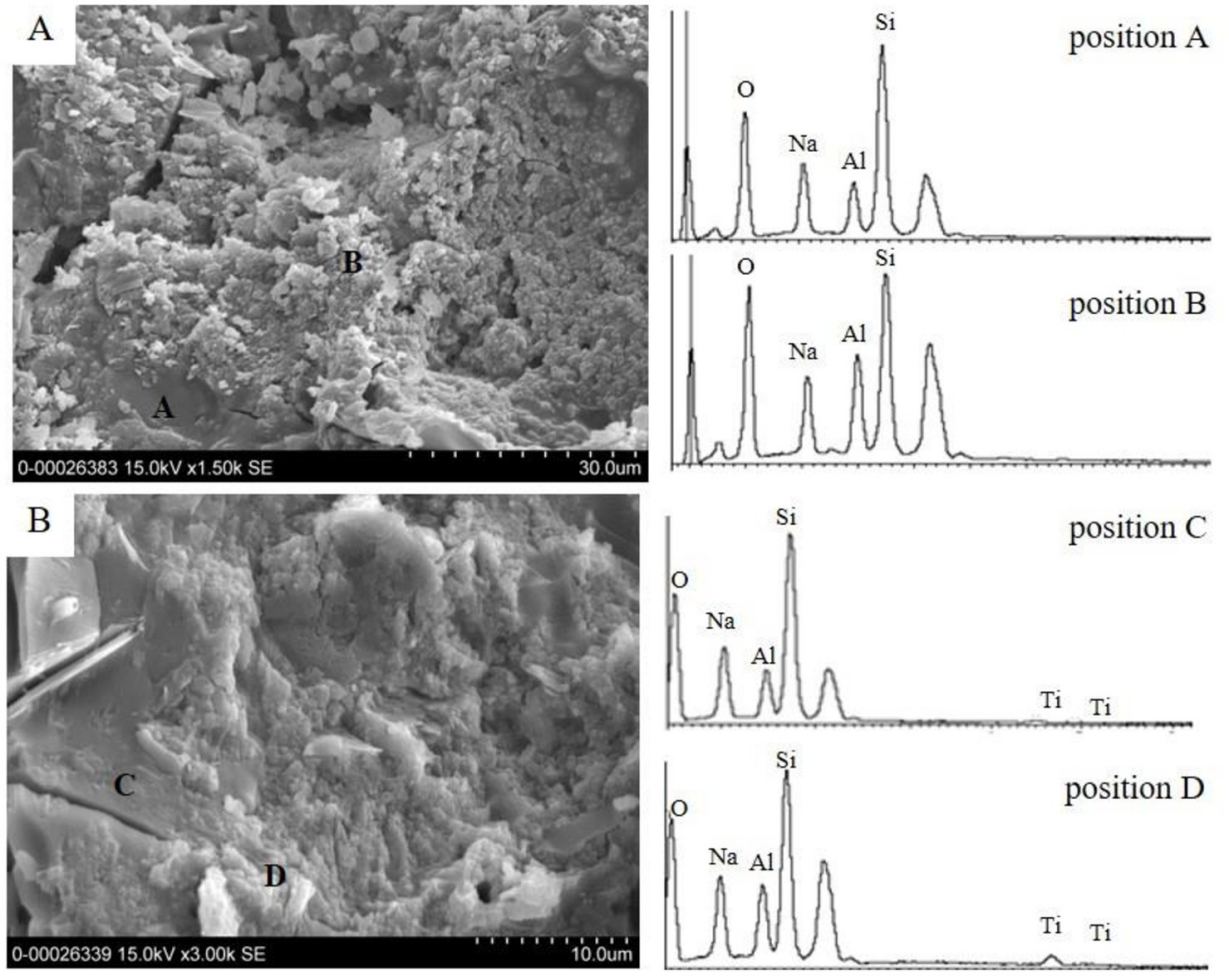
FE-SEM photomicrographs and EDS spectra of MK-based geopolymer synthesized with 40 wt% of (A) zeolite and (B) TiO_2_/zeolite.

The SiO_2_/Al_2_O_3_ molar ratios in the matrices were revealed by EDS (Fig 7). The compositions of the elements and oxides obtained as a result of EDS are summarized in Table 2.

**Table 2:** Compositions of the elements and oxides from EDS in MK-based geopolymer with 40 wt% of (A) zeolite and (B) TiO_2_/zeolite.

Position	Element	(a) Geopolymer + 40%Z		(b) Geopolymer + 40%TZ	
		Element%	Oxide%	Element%	Oxide%
A & C	O	27.60	60.99	36.32	63.09
	Si	17.87	22.50	21.16	20.94
	Al	4.23	5.54	4.73	4.88
	Na	7.13	10.97	9.17	11.09
	Ti			0.66	0.3
	SiO_2_/Al_2_O_3_ molar ratio	4.78		5.07	
B & D	O	29.13	63.78	39.32	54.00
	Si	14.20	17.71	16.93	13.25
	Al	6.68	8.67	6.67	5.31
	Na	6.64	8.67	8.41	8.04
	Ti			2.07	0.91
	SiO_2_/Al_2_O_3_ molar ratio	2.40		2.88	

These results indicated that the matrices of MK-based geopolymer (position A and C) with 40 wt% Z and TZ (Fig 7A and 7B) contained SiO_2_/Al_2_O_3_ molar ratios of 4.78 and 5.07, respectively. These ratios are largely deviated from the SiO_2_/Al_2_O_3_ molar ratio of the starting material (2.25) used for geopolymer preparation. This implied that not all aluminum phases played a role in the development of the geopolymeric structure. In other words, the silicate phase from starting mix was more soluble than the aluminate phases. Therefore, the obtained geopolymeric gel contained a higher SiO_2_/Al_2_O_3_ ratio than the starting mix. The SiO_2_/Al_2_O_3_ molar ratios of the crystalline phases at position B and D for the 40 wt% Z and TZ geopolymers after polymerization were 2.40 and 2.88, respectively. These ratios are slightly different from the SiO_2_/Al_2_O_3_ molar ratio of the starting zeolitic materials (3.0–3.2). Moreover, Ti peaks were observed in both areas of the geopolymeric structure and crystalline particles. The SiO_2_/Al_2_O_3_ molar ratio of the geopolymeric structure obtained by EDS was higher than that of starting mixture. This was in agreement with the residual mineral, such as illite containing Al_2_O_3_ that increased the ratio. As observed by EDS, the SiO_2_/Al_2_O_3_ ratio of the zeolite-geopolymer composite decreased compared to that of the starting zeolitic particles. This observation was attributed to the dissolution of SiO_2_ in the zeolite particles in the presence of a highly alkaline solution.

#### 3.2.3 Molecular structures

**Fig 8:**
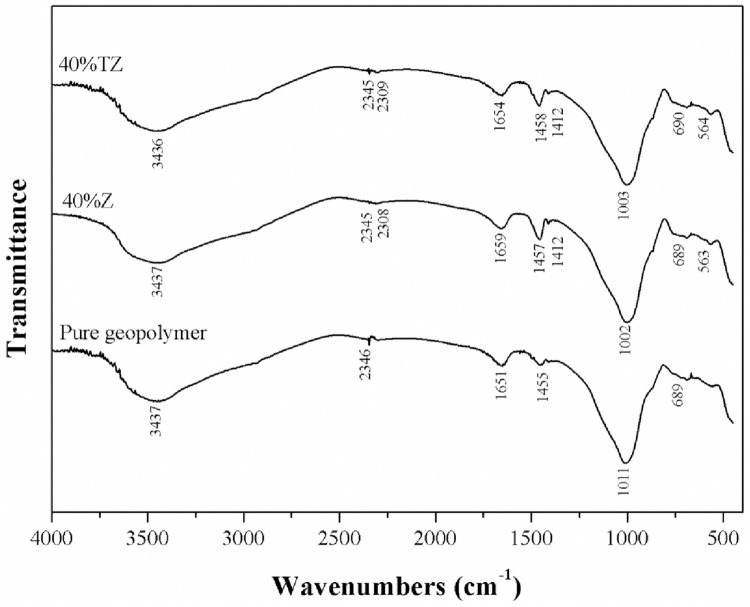
FTIR spectra of MK-based geopolymer with 40 wt% of zeolite and TiO_2_/zeolite.

FTIR spectroscopy, as shown in Fig 8, was employed to investigate the chemical structures of the synthesized MK-based geopolymer with 40 wt% Z and TZ. The broad bands at approximately 3437 cm^-1^ and 1651 cm^-1^ corresponded to the stretching and bending vibration bands, respectively, of the hydroxyl group in the geopolymer composites.

These bands are associated with the weak bonds in H_2_O that were adsorbed on the surfaces or trapped in the cavities of the geopolymers. The absorption band at around 1450 cm^-1^ corresponded to the vibrations caused by the presence of Na_2_CO_3_. The broad band at 990–1050 cm^-1^ was assigned to the internal asymmetric stretching vibration of Si-O-T (T = Si or Al) in the aluminosilicate geopolymeric matrix formed because of the polycondensation or polymerization reaction [43–46]. The adsorption band at 564 cm^-1^ was attributed to the external symmetric stretching of Si-O-T, while that at 689 cm^-1^ to Si-O-Al symmetric stretching and symmetric bending, respectively [29, 45]. By determining and comparing the area under each peak for the pure geopolymer and geopolymers with 40 wt% Z and TZ, it was found that the pure geopolymer possessed the largest area for peaks occurring between 807–1351 cm^-1^ and 2504–3817 cm^-1^, whereas the geopolymer containing 40 wt% TZ displayed the smallest area.

This characterization was carried out in order to verify the stability of the geopolymeric structure in the presence of zeolite and TiO_2_-containing zeolite. For the FTIR spectra of the MK-based geopolymer with 40 wt% TZ, the peak corresponding to Si-O-Ti asymmetric stretching vibration was not detected. Although their charge valency was similar, the replacement of the tetrahedral Si sites in the geopolymeric structure with Ti did not occur in that with TZ. This result confirmed the formation of Ti compounds in a form of TiO_2_ on the zeolite surfaces that afford greater photocatalytic performance [36]. In addition, the pure geopolymer possessed the largest peak areas (807–1351 cm^-1^ and 2504–3817 cm^-1^), implying that this sample had the highest aluminosilicate geopolymeric gel content. The reduction of the peak areas resulted from the addition of 40 wt% of Z and TZ, suggesting a decrease in the geopolymeric gel content.

#### 3.2.4 Compressive strength

**Fig 9:**
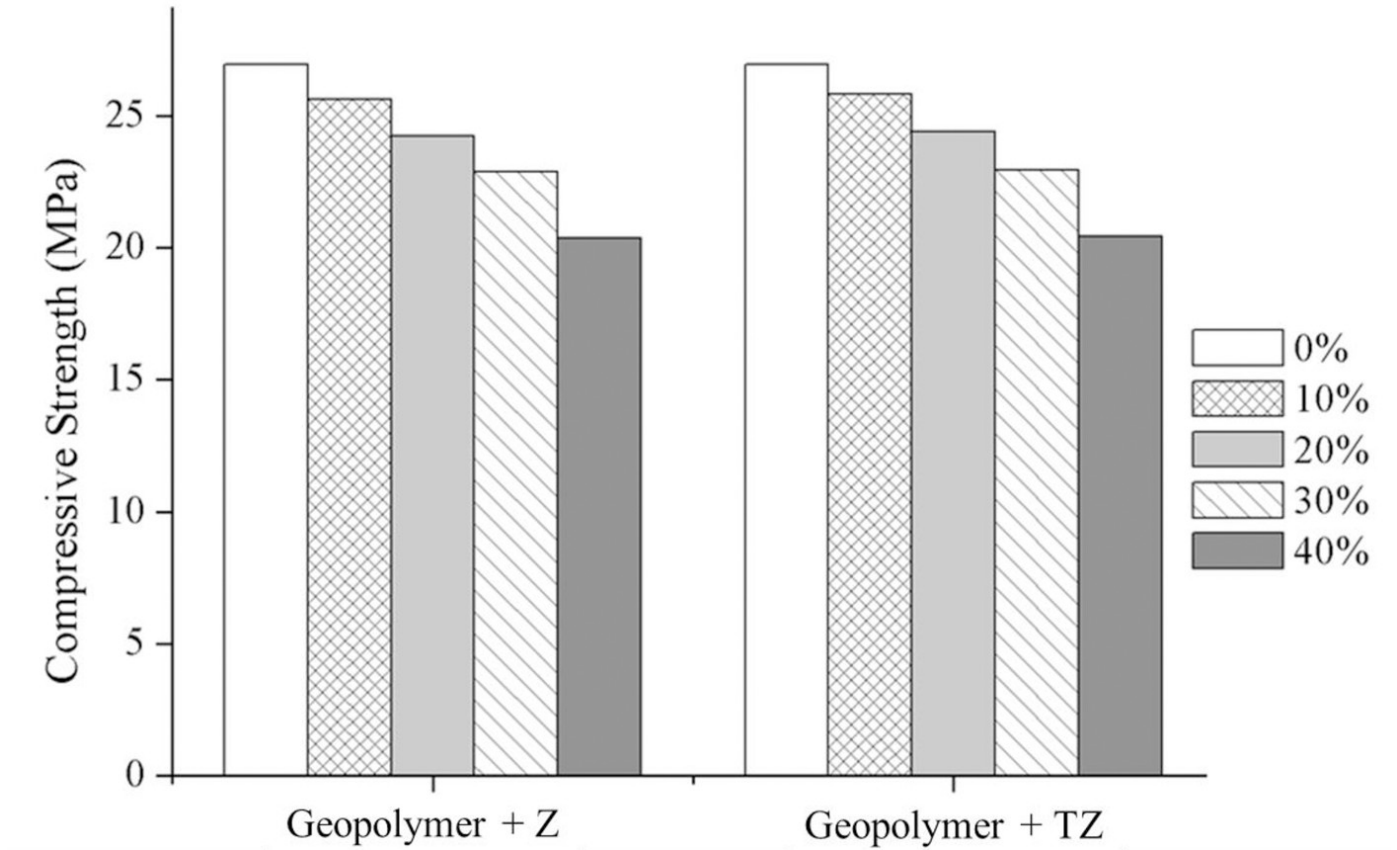
Compressive strength of MK-based geopolymer containing different amounts of zeolite and TiO_2_/zeolite.

The compressive strengths of the MK-based geopolymer with different amounts of Z and TZ are shown in Fig 9. Without Z or TZ, the compressive strengths of the MK-based geopolymer was 26.9 MPa. The strength of the geopolymer decreased with an increase in the amount of Z or TZ from 10 to 40 wt%. These results were in a good agreement with FE-SEM results, which indicate the presence of low-density structures with higher TZ amounts because of the highly porous nature of zeolitic materials [20].

For the geopolymers with 10, 20, 30, and 40 wt% Z, the strengths of geopolymer composites were 25.4, 23.8, 22.8, and 20.3 MPa, respectively. Likewise, the geopolymers containing 10, 20, 30, and 40 wt% TZ, the strengths of geopolymer composites exhibited compressive strengths of 25.6, 23.9, 23.0 and 20.4 MPa, respectively. In addition to the compressive strength measurement, the water absorption of the geopolymer composites with the additions of zeolite and TiO_2_-doped zeolite was determined to evaluate the open porosity of the composites. The water absorption by the pure geopolymer was 1.2%, while that by the geopolymer composites containing 40 wt% TZ and Z were 12.5 and 13.5%, respectively. This confirmed the reduction in the strength of geopolymer composites compared to that pure geopolymer. With higher porosity, the strength decreased. Furthermore, the geopolymer composite that contained 40 wt% TZ displayed lower water absorption than the geopolymer containing 40 wt% Z. This was due to the precipitation of nanosized TiO_2_ on the zeolite surfaces, improving the packing of particles.

### 3.3 Methylene blue removal efficiency and photocatalytic activity

**Fig 10:**
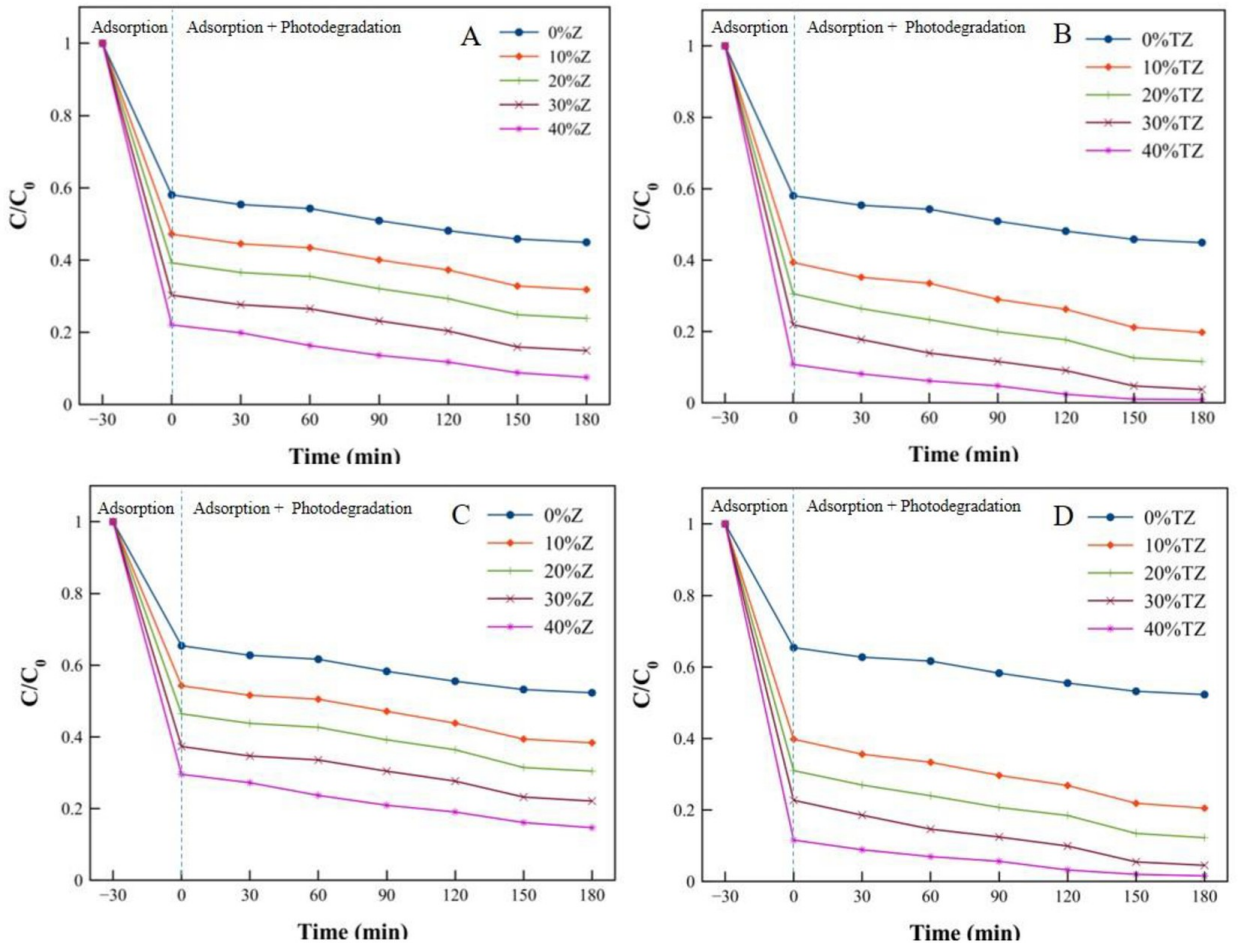
MB adsorption and photocatalytic degradation (at 40 mg/L) of powdered geopolymer composites containing different amounts of zeolite and TiO_2_/zeolite (A) Fresh geopolymer composite containing zeolite, (B) fresh geopolymer composite containing TiO_2_/zeolite, (C) reused geopolymer composite containing zeolite and (D) reused geopolymer composite containing TiO_2_/zeolite.

The MB removal and degradation were evaluated in the dye solution to determine the adsorption capacity and photocatalytic activity of the geopolymer composites, respectively. Before photodecomposition, the geopolymer composite powders were stirred in the MB solution for 30 min at room temperature. This ensured the complete adsorption of MB on the composite surfaces under dark condition, i.e., with no photocatalytic activity. The change in the MB concentration was determined by the variation of the maximum absorbance of MB at a wavelength of 664 nm as a function of the UV light illumination time. Fig 10 shows the MB adsorption and photocatalytic degradation of the powdered geopolymer composites with different amounts of Z and TZ. An apparent change was observed after 30 min under dark conditions; the MB decolorization rate in the dark was higher than that under UV illumination.

It was worth noting here that a remarkably high amount of MB dye was adsorbed because of the high adsorption capacity of faujasite. Faujasite can adsorb MB as well as malachite green dye [47]. Without Z/TZ (0%Z/0%TZ), the adsorption capacity of MK-based geopolymer was lower than that of geopolymers with 10, 20, 30, and 40 wt% Z and TZ. Fig 10A and 10B show a comparison between the Z and TZ amounts. The results revealed that MB decolorization rates at both stages (pure adsorption + photodegradation) for all geopolymers with 10–40 wt% TZ were higher than those for geopolymers containing 10–40 wt% Z. It was worth noting here that without TiO_2_ (Z), the MB degradation for the MK-based geopolymer occurred through the adsorption phenomena, not by photocatalytic process. In the photodegradation process, the degradation of MB by the MK-based geopolymer with TiO_2_-doped zeolite (TZ) was relatively high, and the photocatalytic activity was substantially improved. It is particularly important to maintain cohesion between the support (geopolymeric material) and the catalyst (TZ) during the working time in order to sustain photocatalytic performance. The synthesized geopolymer composites were recovered from the degraded MB dye solution by filtration and reused without pretreatment [47]. Fig 10A and 10C show a comparison between fresh and reused MK-based geopolymers without TiO_2_. The results demonstrated that the adsorption capacity of zeolite which can be reused with no loss in the adsorption capacity. Nevertheless, the zeolite underwent MB adsorption during long-term use. Doping with TZ resulted in a higher adsorption capacity than that of the samples with Z and without Z, as shown in Fig 10B and 10D, respectively, illustrating to regeneration potential of the MK-based geopolymer containing TZ. It should be noted that the MK-based TZ containing geopolymer exhibited high adsorption performance and significantly enhanced photocatalytic degradation activity.

**Fig 11:**
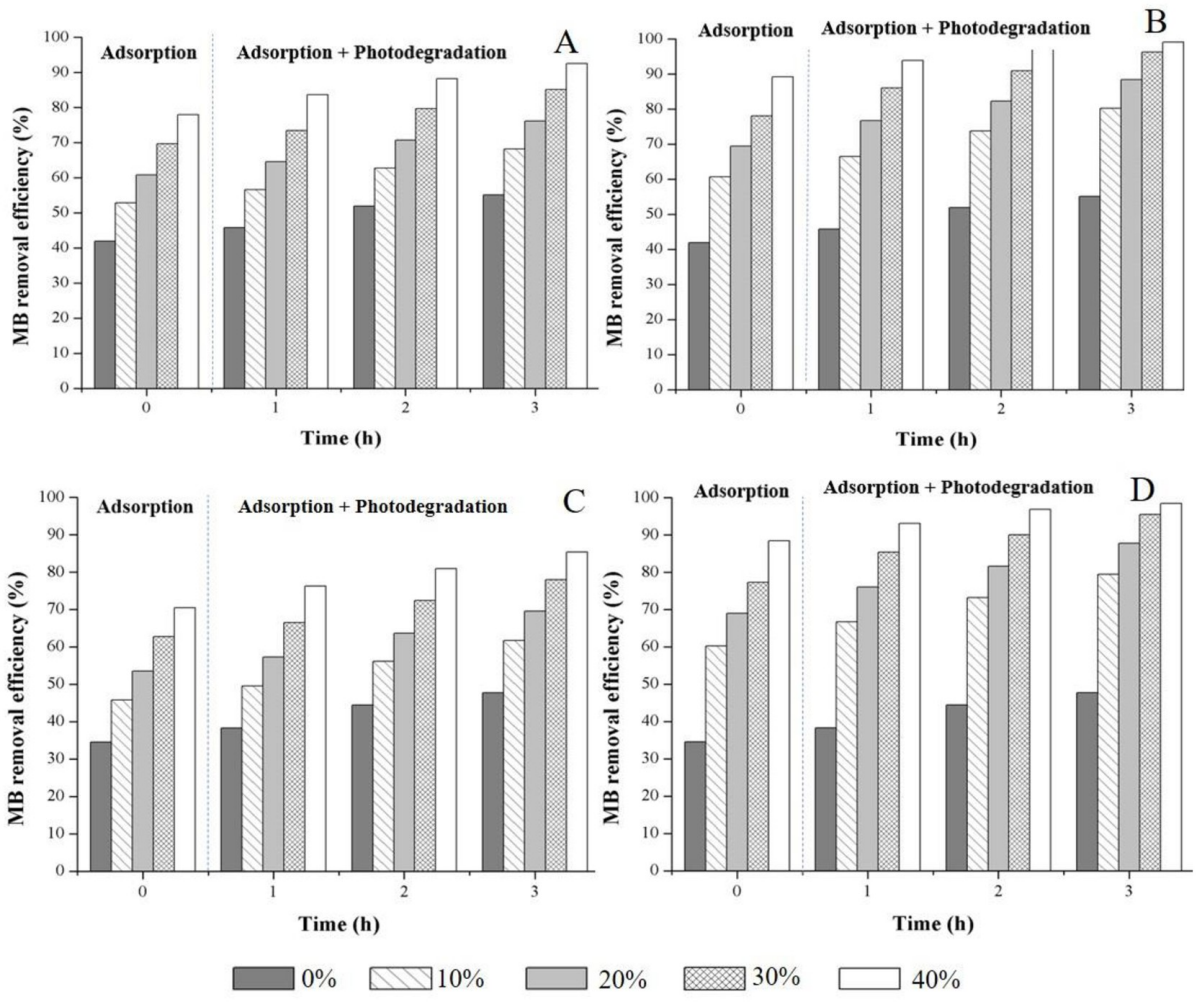
MB removal efficiency (at 40 mg/L) of powdered geopolymer composites containing different amounts of zeolite and TiO_2_/zeolite (A) fresh geopolymer composite containing zeolite, (B) fresh geopolymer composite containing TiO_2_/zeolite, (C) reused geopolymer composite containing zeolite and (D) reused geopolymer composite containing TiO_2_/zeolite.

The percentages of adsorption and adsorption + photodegradation of MB from initial concentrations of 40 mg/L for the MK-based geopolymer with 0–40 wt% of Z and TZ are shown in Fig 11. In the adsorption stage, the MB removal efficiencies of the fresh and reused MK-based geopolymeric materials without Z/TZ were 42.0 and 34.6%, respectively.

The adsorption capacity of reused material decreased. When they were illuminated under UV light for 3h (photodegradation stage), the MB removal efficiencies of the materials without Z/TZ were 55.1 and 47.7%, respectively. For fresh and reused MK-based geopolymeric materials containing 40 wt% Z, the MB removal efficiencies at the adsorption and adsorption + photodegradation stages were 78.0 and 70.5% and 92.5 and 85.4%, respectively. For the fresh and reused MK-based geopolymers with 40 wt% TZ, the MB removal efficiencies at the adsorption and adsorption + photodegradation were 89.2 and 98.4% and 99.1 and 98.4%, respectively. The results suggested that the high adsorption capacity of the geopolymer composites at the adsorption stage can be attributed to the high specific surface area and porosity of the zeolite whereas the excellent photocatalytic activity was caused by the TiO_2_ photocatalyst. Nanosized TiO_2_ plays a critical role in the adsorption stage because the adsorption capacity was independent on the water absorption value. Although the TZ-geopolymer composite possessed a lower water absorption, its MB adsorption was higher than that of the Z-geopolymer composite. MB removal by the geopolymer composites predominantly occurred during the adsorption process and was slightly increased by photodegradation. However, it this composite material retained the support and the catalyst when used repeatedly, without affecting the photocatalytic performance. Compared to the results of our previous study, TZ powder alone displayed MB removal efficiency of 99.4% [36], while 40 wt% TZ-containing composite geopolymer remove MB with an efficiency of 99.1%. Photocatalytic activity of TiO_2_ could be maintained in the same level because of the same size of TiO_2_ particles in both conditions.

**Fig 12:**
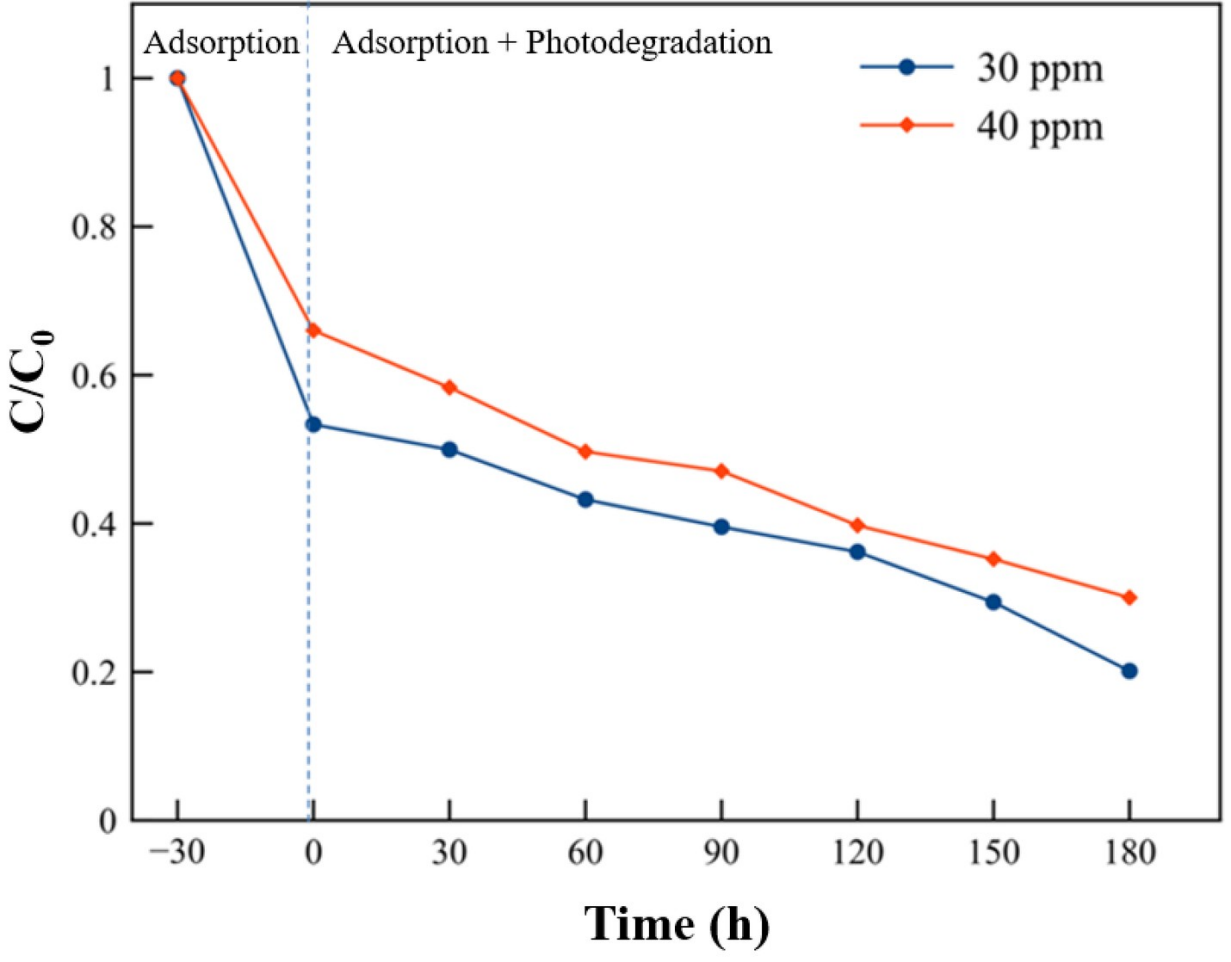
MB adsorption and photocatalytic degradation (at 30 and 40 mg/L) of the geopolymer composite pellet containing 30 wt% TiO_2_/zeolite.

**Fig 13:**
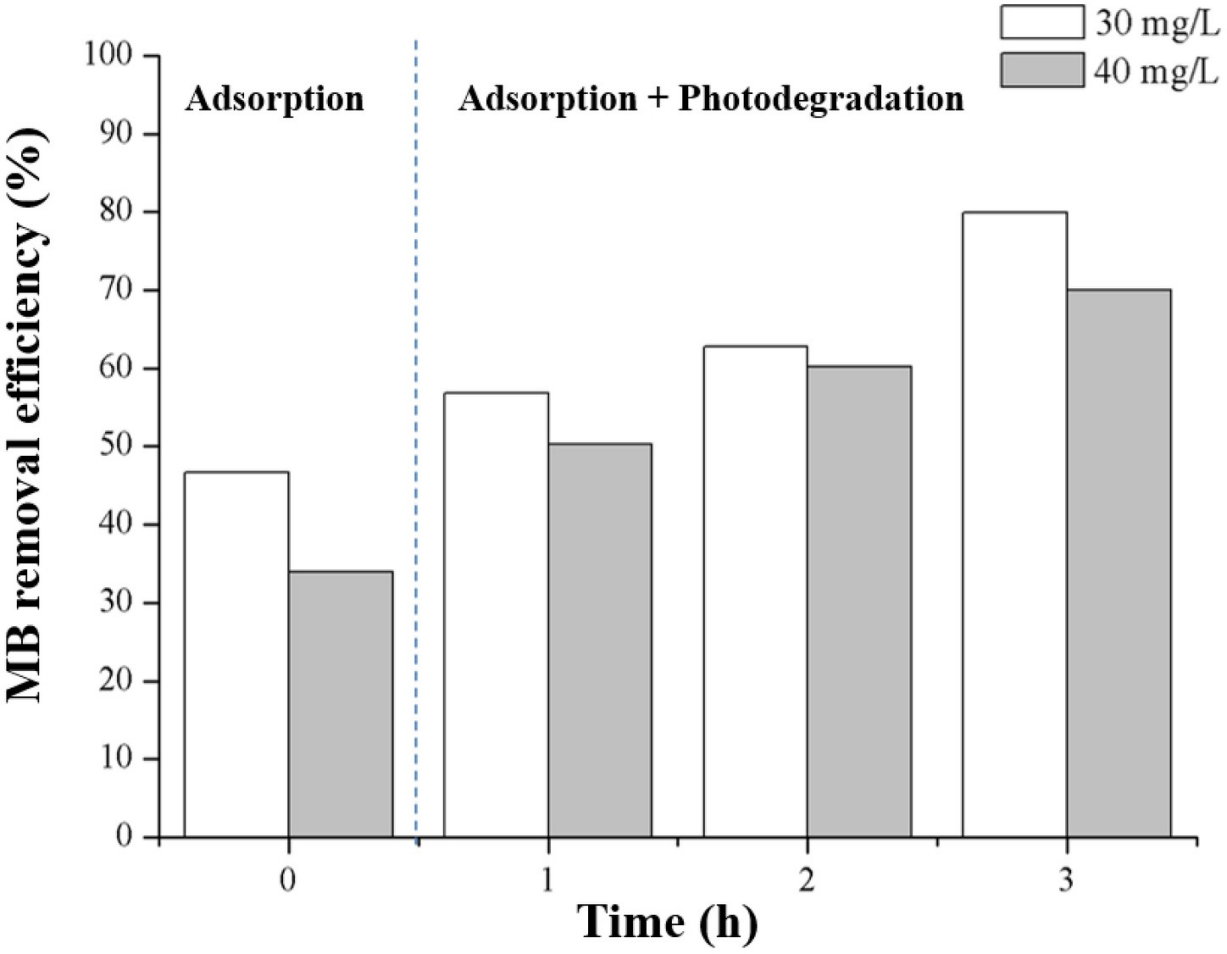
MB removal efficiency (at 30 and 40 mg/L) of the geopolymer composite pellet containing 30 wt% TiO_2_/zeolite.

The MB removal efficiencies during the adsorption and adsorption + photodegradation with initial MB concentrations of 30 and 40 mg/L using MK-based geopolymer pellets containing 30 wt% TZ are depicted in Figs 12 and 13.

As shown in Figs 10B and 11B, the adsorption performance of the geopolymer composite pellet was lower than the powdered geopolymer composite with the same TZ content (30 wt%). The removal percentage of the powdered geopolymer composite was 79% at the adsorption stage, while that of the pellet form was 35% at an initial MB concentration of 40 mg/L. This was due to the difference in the surface area of the samples. During the adsorption and photodegradation stage, the geopolymer composite pellet further removed MB up to 70% efficiency under 3 h illumination. The MB removal efficiency of the powdered geopolymer composite reached 94% of MB removal. The powdered form exhibited advantageous removal over the pelletized geopolymer composites, considering the difference in the quantities used for the experiments. A total of 0.2 and 10 g of the powdered and pelleted geopolymer composites, respectively, were used. However, the pelletized geopolymer composite is easier to handle and collect after use. In addition, the pelletized geopolymer composites can potentially be utilized for environmental cleanup. However, their adsorption and photocatalytic degradation must be improved.

## 4. Conclusion

MK-based geopolymer composites incorporating zeolite or TiO_2_/zeolite were successfully prepared using a SiO_2_/Al_2_O_3_ molar ratio of 2.5 and Na_2_O/Al_2_O_3_ molar ratio of 1.1. The compressive strength of geopolymer composites decreased with increasing amounts of TiO_2_-doped zeolite from 26.9 MPa for 0 wt% to 20.4 MPa for 40 wt%. The composite consists of a geopolymeric material that function as a binder to solidify the zeolite or TiO_2_/zeolite, making the material easy to handle, recover and reuse. The powdered form of the geopolymer composite containing 40 wt% TZ exhibited excellent adsorption capacity and photocatalytic activity, with total MB removal efficiencies of 99.1% and 98.4% for fresh and reused geopolymer composites, respectively. The pelletized geopolymer composite with 30 wt% TZ demonstrated total MB removal efficiency of 70%. The main MB removal mechanism in these composite materials was through adsorption. The photocatalytic activity played a crucial role after the adsorption reached saturation.

## Supporting information

S1 Graphical abstract (DOCX)

## References

[1] Li L, Wang S, Zhu Z. Geopolymeric adsorbents from fly ash for dye removal from aqueous solution. Journal of Colloid and Interface Science. 2006;300: 52–59. 10.1016/j.jcis.2006.03.062 16626729

[2] Khatri A, Peerzada MH, Mohsin M, White M. A review on developments in dyeing cotton fabrics with reactive dyes for reducing effluent pollution. Journal of Cleaner Production. 2015;87: 50–57. 10.1016/j.jclepro.2014.09.017

[3] Hammed AK, Dewayanto N, Du D, Ab Rahim MH, Nordin MR. Novel modified ZSM-5 as an efficient adsorbent for methylene blue removal. Journal of Environmental Chemical Engineering. 2016;4: 2607–2616. 10.1016/j.jece.2016.05.008

[4] Kumar A, Jena HM. Removal of methylene blue and phenol onto prepared activated carbon from Fox nutshell by chemical activation in batch and fixed-bed column. Journal of Cleaner Production. 2016;137: 1246–1259. 10.1016/j.jclepro.2016.07.177

[5] Borges GA, Silva LP, Penido JA, de Lemos LR, Mageste AB, Rodrigues GD. A method for dye extraction using an aqueous two-phase system: Effect of co-occurrence of contaminants in textile industry wastewater. Journal of Environmental Management. 2016;183: 196–203. 10.1016/j.jenvman.2016.08.056 27591846

[6] Yuan W, Yuan P, Liu D, Yu W, Laipan M, Deng L, et al In situ hydrothermal synthesis of a novel hierarchically porous TS-1/modified-diatomite composite for methylene blue (MB) removal by the synergistic effect of adsorption and photocatalysis. Journal of Colloid and Interface Science. 2016;462: 191–199. 10.1016/j.jcis.2015.09.067 26454378

[7] Rida K, Bouraoui S, Hadnine S. Adsorption of methylene blue from aqueous solution by kaolin and zeolite. Applied Clay Science. 2013;83–84: 99–105. 10.1016/j.clay.2013.08.015

[8] Asghar A, Abdul Raman AA, Wan Daud WMA. Advanced oxidation processes for in-situ production of hydrogen peroxide/hydroxyl radical for textile wastewater treatment: a review. Journal of Cleaner Production. 2015;87: 826–838. 10.1016/j.jclepro.2014.09.010

[9] Abu-Danso E, Peraniemi S, Leiviska T, Kim T, Tripathi KM, Bhatnagar A. Synthesis of clay-cellulose biocomposite for the removal of toxic metal ions from aqueous medium. Journal of Hazardous Materials. 2020; 381: 120871–120881. 10.1016/j.jhazmat.2019.120871 31374372

[10] Peláez-Cid AA, Velázquez-Ugalde I, Herrera-González AM, García-Serrano J. Textile dyes removal from aqueous solution using Opuntia ficus-indica fruit waste as adsorbent and its characterization. Journal of Environmental Management. 2013;130: 90–97. 10.1016/j.jenvman.2013.08.059 24071717

[11] Han R, Wang Y, Sun Q, Wang L, Song J, He X, et al Malachite green adsorption onto natural zeolite and reuse by microwave irradiation. Journal of Hazardous Materials. 2010;175: 1056–1061. 10.1016/j.jhazmat.2009.10.118 19954885

[12] Khanday WA, Marrakchi F, Asif M, Hameed BH. Mesoporous zeolite–activated carbon composite from oil palm ash as an effective adsorbent for methylene blue. Journal of the Taiwan Institute of Chemical Engineers. 2017;70: 32–41. 10.1016/j.jtice.2016.10.029

[13] Kuwahara Y, Aoyama J, Miyakubo K, Eguchi T, Kamegawa T, Mori K, et al TiO_2_ photocatalyst for degradation of organic compounds in water and air supported on highly hydrophobic FAU zeolite: Structural, sorptive, and photocatalytic studies. Journal of Catalysis. 2012;285: 223–234. 10.1016/j.jcat.2011.09.031

[14] Pan Z, Stemmler EA, Cho HJ, Fan W, LeBlanc LA, Patterson HH, et al Photocatalytic degradation of 17α-ethinylestradiol (EE2) in the presence of TiO_2_-doped zeolite. Journal of Hazardous Materials. 2014;279: 17–25. 10.1016/j.jhazmat.2014.06.040 25036996

[15] Chen J, Li G, He Z, An T. Adsorption and degradation of model volatile organic compounds by a combined titania–montmorillonite–silica photocatalyst. Journal of Hazardous Materials. 2011;190: 416–423. 10.1016/j.jhazmat.2011.03.064 21501924

[16] Liu S, Lim M, Amal R. TiO_2_-coated natural zeolite: Rapid humic acid adsorption and effective photocatalytic regeneration. Chemical Engineering Science. 2014;105: 46–52. 10.1016/j.ces.2013.10.041

[17] Liu Y, Yu H, Lv Z, Zhan S, Yang J, Peng X, et al Simulated-sunlight-activated photocatalysis of Methylene Blue using cerium-doped SiO_2_/TiO_2_ nanostructured fibers. Journal of Environmental Sciences. 2012;24: 1867–1875. 10.1016/S1001-0742(11)61008-5 23520858

[18] Fukugaichi S, Matsue N. Enhanced optical absorption of nanosized TiO_2_ by composition with zeolite. Materials Chemistry and Physics. 2015;160: 1–4.

[19] Byrappa K, Yoshimura M. Handbook of Hydrothermal Technology: A Technology for Crystal Growth and Materials Processing. William Andrew; 2001.

[20] Takeda H, Hashimoto S, Yokoyama H, Honda S, Iwamoto Y. Characterization of Zeolite in Zeolite-Geopolymer Hybrid Bulk Materials Derived from Kaolinitic Clays. Materials. 2013;6: 1767–1778. 10.3390/ma6051767 28809241 PMC5452502

[21] Juengsuwattananon K, Winnefeld F, Chindaprasirt P, Pimraksa K. Correlation between initial SiO_2_/Al_2_O_3_, Na_2_O/Al_2_O_3_, Na_2_O/SiO_2_ and H_2_O/Na_2_O ratios on phase and microstructure of reaction products of metakaolin-rice husk ash geopolymer. Construction and Building Materials. 2019;226: 406–417. 10.1016/j.conbuildmat.2019.07.146

[22] Xu H, Van Deventer JSJ. The geopolymerisation of alumino-silicate minerals. International Journal of Mineral Processing. 2000;59: 247–266. 10.1016/S0301-7516(99)00074-5

[23] Heah CY, Kamarudin H, Mustafa Al Bakri AM, Bnhussain M, Luqman M, Khairul Nizar I, et al Study on solids-to-liquid and alkaline activator ratios on kaolin-based geopolymers. Construction and Building Materials. 2012;35: 912–922. 10.1016/j.conbuildmat.2012.04.102

[24] Gao K, Lin K-L, Wang D, Hwang C-L, Anh Tuan BL, Shiu H-S, et al Effect of nano-SiO_2_ on the alkali-activated characteristics of metakaolin-based geopolymers. Construction and Building Materials. 2013;48: 441–447. 10.1016/j.conbuildmat.2013.07.027

[25] Davidovits DJ. 30 Years of Successes and Failures in Geopolymer Applications. Market Trends and Potential Breakthroughs. In: Geopolymer 2002 conference. Saint-Quentin (France), Melbourne (Australia): Geopolymer Institute. 2002.

[26] Abdullah MMA, Hussin K, Bnhussain M, Ismail KN, Ibrahim WMW. Mechanism and Chemical Reaction of Fly Ash Geopolymer Cement- A Review. 2011; 10.

[27] Singh B, Ishwarya G, Gupta M, Bhattacharyya SK. Geopolymer concrete: A review of some recent developments. Construction and Building Materials. 2015;85: 78–90. 10.1016/j.conbuildmat.2015.03.036

[28] Hu M, Zhu X, Long F. Alkali-activated fly ash-based geopolymers with zeolite or bentonite as additives. Cement and Concrete Composites. 2009;31: 762–768. 10.1016/j.cemconcomp.2009.07.006

[29] Villa C, Pecina ET, Torres R, Gómez L. Geopolymer synthesis using alkaline activation of natural zeolite. Construction and Building Materials. 2010;24: 2084–2090. 10.1016/j.conbuildmat.2010.04.052

[30] Takeda H, Hashimoto S, Honda S, Iwamoto Y. In-situ formation of novel geopolymer–zeolite hybrid bulk materials from coal fly ash powder. J Ceram Soc Japan. 2010;118: 771–774. 10.2109/jcersj2.118.771

[31] He Y, Cui X, Liu X, Wang Y, Zhang J, Liu K. Preparation of self-supporting NaA zeolite membranes using geopolymers. Journal of Membrane Science. 2013;447: 66–72. 10.1016/j.memsci.2013.07.027

[32] Zhang J, He Y, Wang Y, Mao J, Cui X. Synthesis of a self-supporting faujasite zeolite membrane using geopolymer gel for separation of alcohol/water mixture. Materials Letters. 2014;116: 167–170. 10.1016/j.matlet.2013.11.008

[33] Javadian H, Ghorbani F, Tayebi H, Asl SH. Study of the adsorption of Cd (II) from aqueous solution using zeolite-based geopolymer, synthesized from coal fly ash; kinetic, isotherm and thermodynamic studies. Arabian Journal of Chemistry. 2015;8: 837–849. 10.1016/j.arabjc.2013.02.018

[34] Barbosa TR, Foletto EL, Dotto GL, Jahn SL. Preparation of mesoporous geopolymer using metakaolin and rice husk ash as synthesis precursors and its use as potential adsorbent to remove organic dye from aqueous solutions. Ceramics International. 2018;44: 416–423. 10.1016/j.ceramint.2017.09.193

[35] Bai C, Colombo P. Processing, properties and applications of highly porous geopolymers: A review. Ceramics International. 2018;44: 16103–16118. 10.1016/j.ceramint.2018.05.219

[36] Setthaya N, Chindaprasirt P, Yin S, Pimraksa K. TiO_2_ -zeolite photocatalysts made of metakaolin and rice husk ash for removal of methylene blue dye. Powder Technology. 2017;313: 417–426. 10.1016/j.powtec.2017.01.014

[37] Setthaya N, Chindaprasirt P, Pimraksa K. Preparation of Zeolite Nanocrystals via Hydrothermal and Solvothermal Synthesis Using of Rice Husk Ash and Metakaolin. MSF. 2016;872: 242–247. 10.4028/www.scientific.net/MSF.872.242

[38] Lee S, Jou H-T, van Riessen A, Rickard WDA, Chon C-M, Kang N-H. Three-dimensional quantification of pore structure in coal ash-based geopolymer using conventional electron tomography. Construction and Building Materials. 2014;52: 221–226.

[39] Alwash AH, Abdullah AZ, Ismail N. TiO2-Zeolite Y Catalyst Prepared Using Impregnation and Ion-Exchange Method for Sonocatalytic Degradation of Amaranth Dye in Aqueous Solution. 2013;7: 785–793.

[40] Čižmek A, Subotić B, Aiello R, Crea F, Nastro A, Tuoto C. Dissolution of high-silica zeolites in alkaline solutions I. Dissolution of silicalite-1 and ZSM-5 with different aluminum content. Microporous Materials. 1995;4: 159–168. 10.1016/0927-6513(94)00096-E

[41] Panagiotopoulou Ch, Kontori E, Perraki Th, Kakali G. Dissolution of aluminosilicate minerals and by-products in alkaline media. J Mater Sci. 2007;42: 2967–2973. 10.1007/s10853-006-0531-8

[42] Trochez JJ, Mejía de Gutiérrez R, Rivera J, Bernal SA. Synthesis of geopolymer from spent FCC: Effect of SiO_2_/Al_2_O<3 and Na_2_O/SiO_2_ molar ratios. Mater construcc. 2015;65: e046.

[43] Lee WKW, van Deventer JSJ. Use of Infrared Spectroscopy to Study Geopolymerization of Heterogeneous Amorphous Aluminosilicates. Langmuir. 2003;19: 8726–8734. 10.1021/la026127e

[44] Ferone C, Roviello G, Colangelo F, Cioffi R, Tarallo O. Novel hybrid organic-geopolymer materials. Applied Clay Science. 2013;73: 42–50. 10.1016/j.clay.2012.11.001

[45] Sturm P, Gluth GJG, Brouwers HJH, Kühne H-C. Synthesizing one-part geopolymers from rice husk ash. Construction and Building Materials. 2016;124: 961–966. 10.1016/j.conbuildmat.2016.08.017

[46] Olaremu AG, Adeola AO. Synthesis of faujasite zeolite (Z) for adsorption of cationic dye from textile waste water. International Journal of Modern Research in Engineering and Management. 2018;1: 7–13.

[47] Zainudin NF, Abdullah AZ, Mohamed AR. Characteristics of supported nano-TiO_2_/ZSM-5/silica gel (SNTZS): Photocatalytic degradation of phenol. Journal of Hazardous Materials. 2010;174: 299–306. 10.1016/j.jhazmat.2009.09.051 19818556

