# Supplementary figures and images for: Geopolymer/Zeolite composite materials with adsorptive and photocatalytic properties for dye removal
Source: PLoS One. 2020 Oct 30;15(10):e0241603. doi: 10.1371/journal.pone.0241603 (PMC7598482; doi:10.1371/journal.pone.0241603)

**S1 Graphical abstract.**


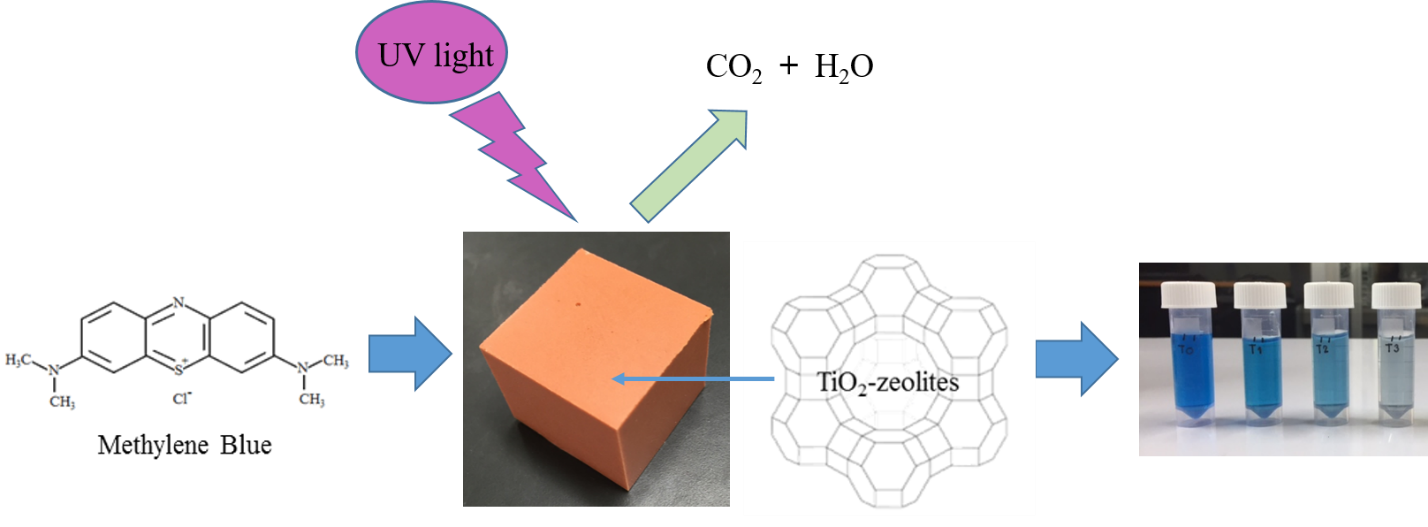

Supplement: S1 Graphical abstract — (DOCX) [file pone.0241603.s001.docx]
